# Ultrasound‐assisted extraction of boulardii yeast cell wall polysaccharides: Characterization and its biological functions on early‐weaned lambs

**DOI:** 10.1002/fsn3.2318

**Published:** 2021-05-13

**Authors:** Mengjian Liu, WuJun Liu, WenJu Zhang, Jun Yao, Xucheng Mo

**Affiliations:** ^1^ College of Animal Science and Technology Shihezi University the North 4 Road Shihezi Xinjiang 832003 China; ^2^ College of Animal Science Xinjiang Agriculture University Urumuqi Xinjiang 830000 China

**Keywords:** cell wall polysaccharides, diarrhea, feed conversion, ileum morphology, jejunum morphology, microbiota, response surface methodology, *Saccharomyces* *boulardii*

## Abstract

Firstly, this study was designed to determine the optimal ultrasound‐assisted extraction parameters of *Saccharomyces* *boulardii* yeast wall polysaccharides (BYWP). Besides, the molecular weight and the ratio of mannose to glucose in compositions of BYWP were determined. Also, the effects of BYWP on the gain feed ratio, diarrhea frequency, intestinal morphology, intestinal immunity, and intestinal microbial flora of early‐weaned lambs were investigated. Single‐factor tests and Response surface optimization analysis (RSA) were used to obtain the optimal ultrasound‐assisted extraction conditions. Sephadex G‐100 column chromatography and liquid chromatography were used to analyze the molecular weight and ratio of mannose to glucose. The feeding trial was used to observe the biological functions of BYWP on early‐weaned lambs. A total of 72 36‐day‐old crossbred early‐weaned lambs were randomly divided into 4 groups with 3 replicates per group and 6 lambs per replicate. Lambs in the four treatments were fed basal milk replacer without supplement (Group I), basal milk replacer+0.1% BYWP (Group II), basal milk replacer+0.3% BYWP (Group III), and basal milk replacer+0.5% BYWP (Group IV), respectively. The optimal ultrasound‐assisted extraction parameters were as follows: NaOH addition: 52.63%, ultrasonic power: 143.15 W, ultrasonic time: 86.20 min, and the optimized extraction yield reached 37.54%. The molecular weights of main components BLC‐1 and BLC‐2 were 164.68 KDa and 13.21 KDa, and their proportions in BYWP were 24.57% and 66.08%, respectively. The proportions of glucose, mannose in BLC‐1 and BLC‐2 were 47.68%, 39.18%, and 76.59%, 6.86%, respectively. The addition of 0.3% and 0.5% BYWP in basal milk replacer significantly increased the average daily gain and feed conversion rate, and decreased the average fecal index and diarrhea rate of early‐weaned lambs. The addition of 0.3% and 0.5% BYWP significantly enhanced the intestinal morphology (villus height, crypt depth, and V/C value) of jejunum, ileum (*p* < .05). The addition of 0.3% and 0.5% BYWP significantly improved the levels of SIgA and IL‐10, but significantly decreased the level of IL‐1 in the ileum (*p* < .05). The addition of 0.3% and 0.5% BYWP significantly increased the number of *Lactobacillus*, but significantly suppressed the growth of *Salmonella* and Clostridium *perfringens* (*p* < .05). The results of the present study suggest that the supplementation of BYWP in the diet of early‐weaned lambs could increase feed utilization rate, and enhance intestinal morphology, immunological competence, microbial flora balance, and decrease the rate of diarrhea occurrence.

## Graphical abstract

The optimal ultrasound‐assisted extraction parameter for BYWP was confirmed. The molecular weight of main components of BYWP were tested. The effects of BYWP on intestinal morphology, intestinal immune, intestinal microflora of early‐weaned lambs have been confirmed.

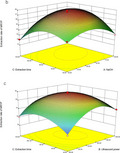

## 1 INTRODUCTION

*Saccharomyces boulardii* yeast wall polysaccharides (BYWP) is a natural prebiotic, which is one of the main components of the bioactivity of *Boulardii* yeast. The main components of BYWP are β‐glucan (40%–60%), mannosan (30%–40%), and chitin (2%–10%) (Chen et al., 2018). Recently, BYWP has been regarded as a feed supplement for reducing the diarrhea rate and mortality on young animals in the local pasture. Some reports indicated that yeast wall polysaccharides, such as *Boulardii* yeast, possess a lot of physiological functions, such as immunity‐boosting properties, regulation of intestinal microbiota balance eliminating pathogenic bacteria, improved nutrient utilization, the adsorptive activity of toxins, and better health status of ruminants (Muthusanmy et al., 2011; Mahesh et al., 2020). As it is well known that the molecular weight and monosaccharide compositions are fundamental indicators, which could be investigated to imply the polysaccharides bioactivity (Cordero et al., 2011).

Various extraction techniques could be used to produce the pure BYWP, such as conventional solid‐liquid extraction, Soxhlet extraction, and percolation. But low extraction efficiency, toxic solvent residue in the extract, and losses of polysaccharides compounds might be encountered using these extraction methods (Khadhraoui, 2017). Therefore, an appropriate extraction method for BYWP is crucial to be found. Ultrasound‐assisted extraction (UAE) of natural products is a high‐efficiency “environment friendly” method in the 21st, and UAE could protect the environment and in the meantime enhance the competitiveness of industries to more economic, ecologic, and innovative (Sicaire et al., 2016). Compared with the traditional extraction process, UAE could reduce the consumption of solvents, shorten the extraction time, and enhance the extraction efficiency with a higher level of automation (Wang et al., 2007). More importantly, ultrasound‐assisted extraction does not induce a huge decrease or change in molecular structure (Rostagno et al., 2003). The mechanism of UAE is that the acoustic cavitation in UAE could disrupt yeast cell walls, reduce the particle size and enhance the connection between targeted compounds and solvents (Farid et al., 2020). The extraction parameters such as extraction time, temperature, pH, and ultrasonic power can significantly influence the yield. At present, UAE has been used to extract the polysaccharide and oligosaccharide compounds from plant and microorganisms in many areas (Hromádková and Ebringerová, 2003; Tor and Aydin, 2006; Živković et al., 2018). Therefore, it is essential to optimize the appropriate extraction parameters which enable good yield, and a high level of bioactive compounds.

For shortening the breeding cycle of ewes and improving the productivity by increasing the frequency of lambing, the early‐weaned lambs are usually artificially compulsorily weaned at 35‐day‐old, which resulted in an incredibly high rate of diarrhea and mortality, especially in the changeover period between winter and spring in the pasturing area in Xinjiang (Dongshan et al., 2009). We have to find a natural feed supplement to replace the antibiotics, which have been prohibited in the lamb industry.

Therefore, we investigated the effects of ultrasonic time, NaOH addition, and ultrasound power on the ultrasound‐assisted extraction yield of BYWP by a method of a Single‐factor experiment. Thereafter, we determined the optimal ultrasound‐assisted extraction parameters of BYWP by Box–Behnken design in response surface methodology (RSM). Besides, we analyzed the molecular weight and the ratio of mannose and glucose of BYWP components by Sephadex G‐100 column chromatography and liquid chromatography. Finally, the effects of addition of 0.1%, 0.3%, and 0.5% BYWP on feed to gain ratio (F/G), the incidence of diarrhea, intestinal morphology, cytokines expression, and gut microbes indices were examined in a feed trail of early‐weaned lambs. This research has not been reported until now, and it could provide a fundamental UAE condition for pure BYWP form *Saccharomyces* *boulardii* yeast in industry, and also provide a theoretical basis for the new replacement for antibiotics in early‐weaned lamb.

## 2 MATERIALS AND METHODS

### 2.1 Experimental strain

The experimental strain of *Boulardii* yeast was obtained from the College of Animal Science and Technology, Shihezi University, Shihezi, China.

### 2.2 Single‐factor experiments of UAE parameters

The UAE parameters of extraction time, NaOH addition, and ultrasound power were used as a single factor in this study, respectively. The fundamental UAE parameters were as follows: extraction time 80 min, NaOH addition 40 g/L, and extraction power 100 W. Three UAE extraction factors were as follows: The extraction time was tuned to 0, 40, 80, 120, 160, and 200/min; the NaOH addition was tuned to 0, 20, 40, 60, 80, and 100/ g/L; the ultrasound power was tuned to 0, 50, 100, 150, 200, and 250/W. Each process was repeated three times. The ranges of 3 UAE independent parameters have been confirmed.

### 2.3 Box–Behnken design of UAE parameters

Box–Behnken designs (BBD) are a class of rotatable or nearly rotatable second‐order designs based on three‐level incomplete factorial designs. BBD matrix is slightly more efficient than the central composite design but much more efficient than the three‐level full factorial designs where the efficiency of one experimental design. Another advantage of the BBD is that it does not contain combinations for which all factors are simultaneously at their highest or lowest levels. So these designs are useful in avoiding experiments performed under extreme conditions, for which unsatisfactory results might occur. So we choose the BBD design to confirm the most optimized UAE parameter (Ferreira et al., 2007).

Based on the result of the Single‐factor experiment, the response surface optimization models of three factors and three levels were implemented, and the response variables represented the extraction yield of polysaccharides (Table 1). Triplicate yield verification experiments were conducted to verify the availability and accuracy of the mathematical model. Moreover, the relative error between the theoretical and actual yield was calculated (Liu et al., 2014).

**TABLE 1:** Independent variables and levels in Response surface optimization models

	A	B	C
Coding value	NaOH addition/ g/L	Ultrasound power/W	Extraction time/min
−1	40	100	40
0	60	150	80
1	80	200	120

### 2.4 Determination of the molecular weight and monosaccharide compositions

After the procedures of purification and separation, the molecular weight of BYWP segments was analyzed by high‐performance gel permeation chromatography in an Agilent 1,200 HPLC system, which was installed with a TSKgel‐G3000‐PWXL (7.8 mm×300 mm) column. The operation parameters were as follows: column temperature was 85 ℃; mobile phase was deionized water; flow velocity was 0.5 ml/min; and the sample size was 50 μL (Sun et al., 2009). Standard dextrans (2.5 kDa, 5.9 kDa, 11.8 kDa, 22.8 kDa, 47.3 kDa, 112 kDa, 212 k Da, and 404 kDa) were used for obtaining the standard curve (Guo et al., 2010). Besides, according to the analysis processes reported by Jin M, the proportions of mannose and glucose in BLC‐1 and BLC‐2, which were defined as two main BYWP compositions, were determined by HPLC with a TSKgel‐G3000‐PWXL (7.8 mm×300 mm) column (Jin et al., 2012).

### 2.5 Experimental animals and experimental design

This experiment was arranged as a single factor randomized complete block design. A total of 72 crossbred early‐weaned lambs (48 male and 24 female lambs, Kazak♂ *Altay♀ * Suffolk♂) weighing 7kg (36‐days age) were blocked by sex and weight and assigned to one of four dietary treatments with three replicates consisting of six lambs (four males and two females per replicate pen) per treatment. All experimental lambs were compulsorily weaned at 36‐day‐old. Lambs in the four treatments were fed basal milk replacer without supplement (Group I), basal milk replacer+0.1% BYWP (Group II), basal milk replacer+0.3% BYWP (Group III), and basal milk replacer+0.5% BYWP (Group IV), respectively. The milk replacer was formulated to meet the nutrient requirement of 8–25 kg lambs with the body weight gain of 300 g/day recommended by the Chinese Feeding Standard of Lamb (2007) (Table 2).

**TABLE 2:** Ingredient compositions and chemical analysis of milk replacer (air‐dry basis) %

Items	Content	Nutrient levels	Content
Ingredients		Nutrition level	
Expended corn	41.50	Dry matter	90.67
Alfalfa hay	8.00	Digestible energy DE/(MJ/kg)	17.38
Soybean oil	2.00	Crude protein	23.75
Expended soy	16.00	Crude fat	16.08
Fermented Soybean	20.00	Neutral detergent fiber	4.81
Premix	1.00	Calcium	0.53
Baby Formula Milk Powder	10.00	Phosphorus	0.41
NaCl	0.30	Lysine	0.91
CaHCO3	1.00	Methionine +cysteine	0.61
NaHCO3	0.20	Threonine	0.65
Total	100.00	Concentrate: roughage	90:10

Note:

Without adding antibiotics in the ingredients. The nutrient levels were calculated values. The premix provided the following per kg of the milk replacer:: Fe (as ferrous sulfate), 20 mg; Zn (as zinc sulfate), 35 mg; Cu (as copper sulfate), 6 mg; Mn (as manganese sulfate), 25 mg; I (as potassium iodide), 0.25 mg; Se (as sodium selenite), 0.2 mg; Co (as cobalt sulfate), 0.1 mg; Vitamin A,9,000 IU; Vitamin D,1,050 IU; Vitamin E,28 IU; Vitamin B1,3.0 mg; Vitamin B2,2.4 mg; Vitamin B6,7.0 mg; Vitamin B12, 0.02 mg;VK3, 1 mg; biotin, 0.08 mg; folic acid, 0.90 mg; D‐pantothenic acid, 10 mg; nicotinic acid, 12 mg. The nutrient levels were calculated values by methods of Chinese Standard GB/T. Roughage was comprised of radix astragali, notoginseng radix, ligustici, red dates, wormwood, and alfalfa followed feed formula by the farm management.

#### 2.5.1 Effect of BYWP on the feed conversion ratio of early‐weaned lamb

The feeding trial lasted 32 days. Feed intake per lambs was recorded daily. Lambs were weighed individually at the start and end of the feeding trial (Yoo et al., 2018). Feed intake and feed conversion ratio (F/G) were calculated as following formulations:

F/G= [average daily feed intake/ average weight gain] ×100%

#### 2.5.2 Effect of BYWP on diarrhea incidence of early‐weaned lamb

A hierarchical system of the average fecal score was conducted to present the severity extent of diarrhea (Table 3). In the whole feeding trial, all experimental lambs were observed for clinical signs of diarrhea individually and recorded twice daily (08:00 and 15:00). Diarrhea was recorded, if a lamb presented a fecal score>3, and for 3 consecutive days (Melin et al., 2010). At the end of the feeding trial, the diarrhea frequency and average fecal score were counted as the following formulations:

**TABLE 3:** Fecal samples score of lamb feces

Score	Trait
1	hard firm feces; without fetid odor
2	slightly soft feces; without fetid odor
3	soft, and partially formed feces; with a light fetid odor
4	loose, semi‐liquid feces (diarrhea); with a distinct fetid odor
5	watery, mucous‐like feces (severe diarrhea); with a pungent fetid odor

Diarrhea frequency (%) = (diarrhea days×the number of diarrhea lambs) / (experimental days×the total number of lambs) * 100%.

Average fecal score (%) = Sum of fecal scores /the total number of lambs.

#### 2.5.3 Sample collection and preparation

At the end of the feeding trial, three experimental lambs (two males and one female) in each pen were randomly selected and euthanized and dissected. The segments (3–5 cm) of jejunum and ileum were taken out and rinsed by 0.9% sodium chloride solution both inside and outside (Xu et al., 2018). The rinsed jejunum and ileum segments were randomly divided into 2 groups. One group of segments was put into bottles, which filled with 10% formalin for observation of intestinal morphology (Wang et al., 2012). Another group of segments was put into aseptic bags and stored in a liquid nitrogen jar for analysis of mucosa cytokines and antibodies. The ileum segments with contents were put into aseptic bags, which filled with cryoprotectants (5% dimethyl sulfoxide +10% glycerin +culture medium) and stored in the liquid nitrogen jar for analysis of intestinal microflora (Keelan et al., 1985).

#### 2.5.4 Determination of immune parameters

Commercial sheep‐specific ELISA kits (AmyJet Scientific Inc, Wuhan, China) were used to quantify the immunoglobulin (SIgA, IgA, and IgG), the serum cytokines (interleukin (IL)–1, IL‐10), and tumor necrosis factor TNF‐α according to the manufacturer's instructions.

#### 2.5.5 Determination of intestinal microflora in ileum content

In this experiment, all processes were carried out in a sterile condition. 2 g contents of ileum segments were weighted. The number of *Bifidobacterium*, *Lactobacillus*, *Escherichia coli*, *Salmonella,* and *Clostridium* *perfringens* was counted by the dilution method of plate counting. The culture mediums and conditions were shown in Table 4.

**TABLE 4:** Culture medium and culture conditions of different bacteria

Items	mediums	Culture condition
Bifidobacterium	BBL: Agar medium	Anaerobic, 37℃, pH=7,48h (Peng and OU, A. F., 2014)
Lactobacillus	MRS: Man rugosa and sharpe medium	Anaerobic, 42℃,p H = 6.3,72h (Lee et al., 2016)
Escherichia coli	EMB: Eosin methylene blue agar	Aerobiosis, 37℃, pH=7.2,48h (Jin et al., 2001)
Salmonella	HE: Hektoen enteric agar	Aerobiosis, 37℃, pH=7.0,48h (Gieraltowski et al., 2016)
*Perfringens*	SPS Sulfite polymyxin sulfadizine agar	Anaerobic, 37℃, 48h (Xie, 2017)

### 2.6 Statistical analysis

All raw data were preliminarily recorded and calculated by EXCEL (Microsoft office version 2019 for Windows, Microsoft Inc., Chicago, IL, USA). The statistical values and significant differences among the means were statistically analyzed and represented as mean±standard deviation by using one‐way analysis of variance (ANOVA) by Duncan's test of SPSS software (version 18.0 for Windows, SPSS Inc., New York, IL, USA). Design‐Expert software (version 8.0.6, State Ease Inc., Minneapolis, NE, USA) was used to analyze the variance (ANOVA) and the parameters of the response equation.

## 3 RESULTS

### 3.1 Effect of independent variables on BYWP extraction yield

**FIGURE 1:**
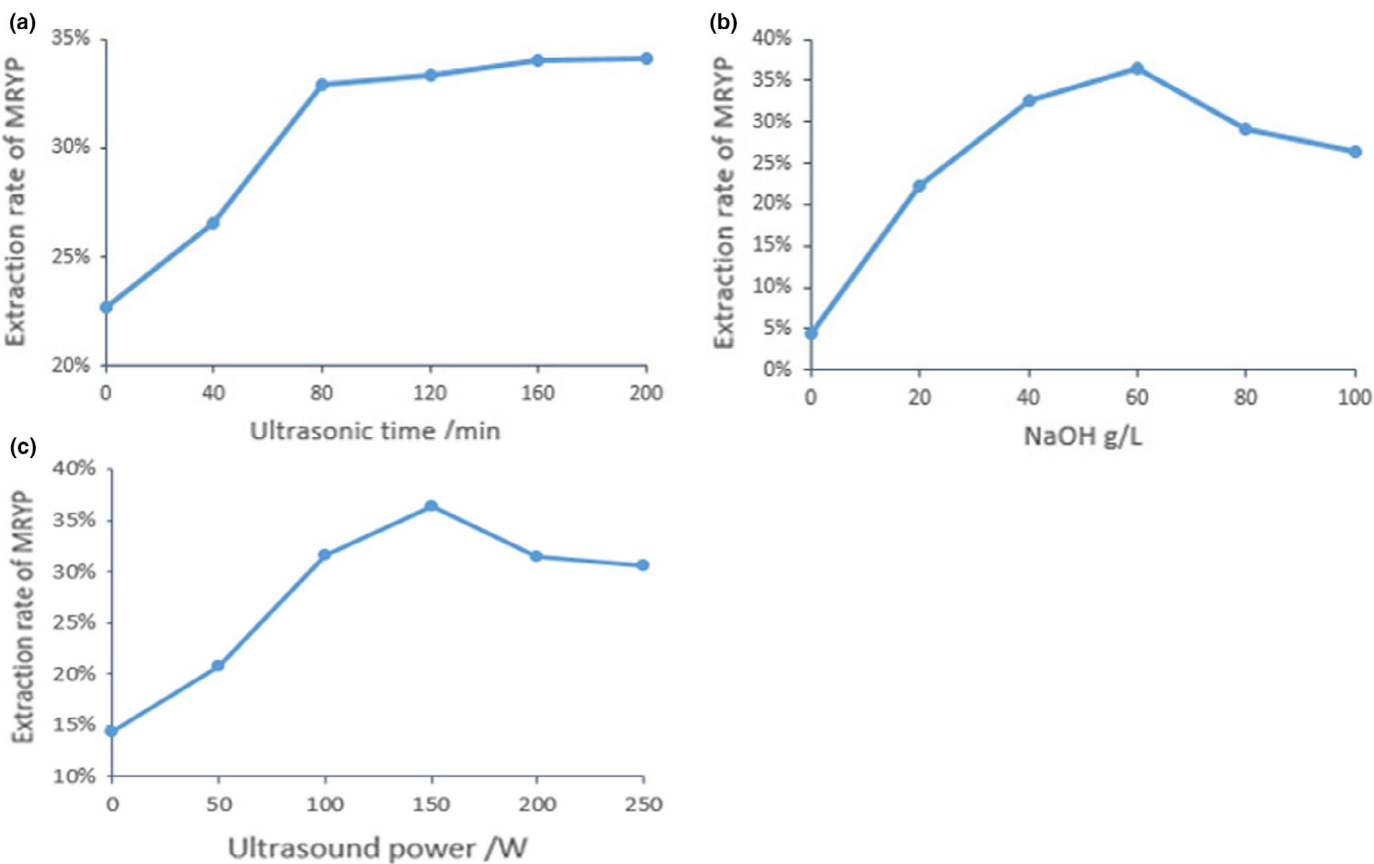
Effects of different extraction parameters on yield of BYWP ultrasonic time (a), NaOH addition (b), and ultrasonic power(c)

As shown in Figure 1a, the extraction yield of BYWP increased rapidly when the ultrasonic time from 30 to 80 min, but there was no longer an obvious change in the extraction yield as the ultrasonic time continued to increase. Finally, it was being proved by experiments that it consumed too much extra energy for little changed extraction yield after an ultrasonic time of 80 min. Therefore, we decided that 40–120 min was the suitable range of ultrasonic time in ultrasonic extraction. Figure 1b showed the effect of NaOH addition on the extraction yield of BYWP. The results showed that the extraction yield of BYWP increased significantly as NaOH addition increased, and then decreased when the NaOH addition was over 60 g/L. The highest BYWP yield was 36.24% when NaOH addition was 60 g/L. Therefore, the proper range of NaOH addition was 40, 60, and 80 g/L. As shown in Figure 1c, the ultrasound power displayed a positive linear effect on the extraction yield of BYWP when the ultrasound power from 0 to 150 W, and then the extraction yield decreased as ultrasound power increased. The pick of extraction yield was 36.41% when ultrasound power was 150 W. So we decided that the ultrasound power scope of 100, 150, and 200 was used as the proper range in the extraction yield of BYWP.

### 3.2 Optimization of BYWP extraction yield by response surface methodology

In this study, the Response surface optimization methodology (RSM) was conducted for precise optimization parameters. All 17 random sequential tests under various ultrasonic extraction parameters were performed to analyze the reciprocal influence of independent variables (NaOH addition, ultrasonic power, and ultrasonic time) on BYWP extraction yield.

The following parameters were adopted in this Box–Behnken design: NaOH addition (A) 40, 60, 80/g/L. ultrasonic power (B) 100, 150, 200/W and ultrasonic time (C) 40, 80, and 120 min (Table 5). The extraction yield of BYWP reached its peak value of 36.89%, when extraction parameters as NaOH addition 60/ g/L, ultrasonic power 150 W, ultrasonic time 80 min (*p* <.05). The final second‐order polynomial equation obtained in terms of actual factors was given as below:

**TABLE 5:** BBD matrix and response values for ultrasound‐assisted extraction yield of BYWP

RUN	A (g/L)	B (W)	C (min)	Extraction yield of BYWP (%)
1	60	100	120	32.07
2	80	150	120	34.84
3	60	150	80	30.01
4	60	200	40	33.84
5	60	150	80	30.09
6	80	150	40	33.10
7	60	100	40	30.86
8	60	150	80	35.62
9	80	100	80	32.84
10	40	150	40	34.27
11	60	150	80	35.25
12	60	200	120	33.48
13	60	150	80	34.50
14	40	150	120	32.13
15	40	100	80	31.57
16	80	200	80	34.01
17	40	200	80	32.04

(Y)=−44.3532 + 0.1831 × A+0.096954 × B+1.770625 × C+0.000106×AB+0.0061×AC‐0.0002×BC‐0.00425 × A^2–7.4E‐0.5 × B^2–0.02001 × C^2.

This model was highly statistically significant because of a high model *F*‐value (73.408) and a low P‐value (*p* <.0001). The high R_2_ value of 0.9895 and the high R_2adj_ value of 0.9760 indicates that more than 98.98% of the response variability was explained, and supporting ability and accuracy of the established. The C.V was 1.0896% in this model, which indicated that the simulation was reasonably reproducible. The Lack of Fit and the comparison of pure error were insignificant because the *F*‐value was 4.3338 (*p* >.05), which indicated that the model was accurate (Table 6).

**TABLE 6:** Analysis of variance (ANOVA) for the fitted quadratic polynomial model for optimization of BYWP production by ultrasound‐assisted extraction

Item	Quadratic sum	Degree of freedom	Mean square	*F*‐value	P‐value
Model	92.79563	9	10.31063	73.408	< 0.0001
A‐NaOH	5.74605	1	5.74605	40.90984	0.0004
B‐Ultrasound power	6.177613	1	6.177613	43.98241	0.0003
C‐Extraction time	0.262813	1	0.262813	1.871132	0.2136
AB	0.7225	1	0.7225	5.143944	0.0576
AC	5.9536	1	5.9536	42.38752	0.0003
BC	0.616225	1	0.616225	4.387304	0.0745
A^2	12.14338	1	12.14338	86.45657	< 0.0001
B^2	37.15939	1	37.15939	264.5617	< 0.0001
C^2	16.85474	1	16.85474	119.9998	< 0.0001
Residual	0.983195	7	0.140456		
Lack of Fit	0.751875	3	0.250625	4.333823	0.0953
Pure Error	0.23132	4	0.05783		
Cor Total	93.77882	16			
R^2^	0.9895	Adeq Precision	0.9760		
R^2^_adj_	0.9760	C. V%	1.0896		
R^2^_pred_	0.8679	r	0.9678		

Note:

*p* <.05, significant difference; *p* <.01,very significant difference.

**FIGURE 2:**
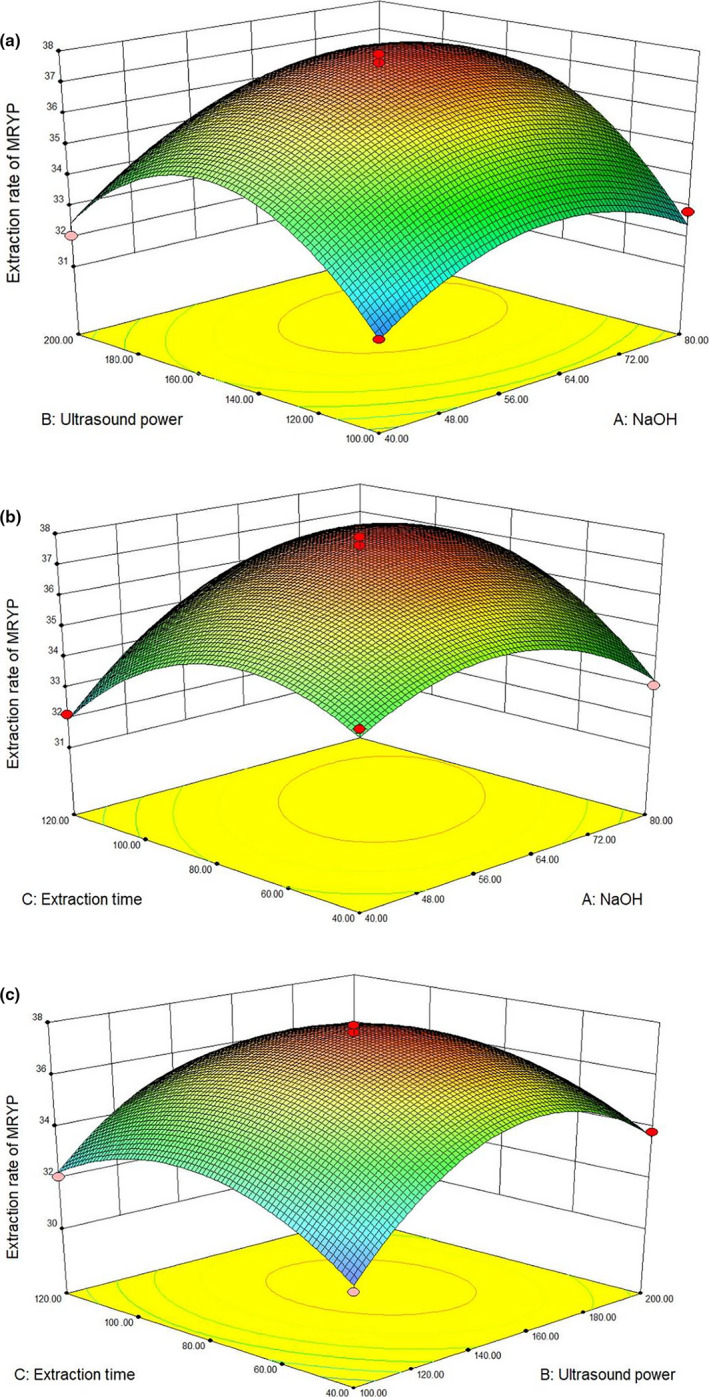
The 3D‐plot and 2D‐projection of response surface represent the interaction between two factors in BYWP production (µg/L) by keeping the other two variables constant: ultrasound power and NaOH addition (a), ultrasound time and NaOH addition (b), ultrasound time and ultrasound power (c), (the yellow and red dot represent design points below predicted value and above predicted value, respectively)

As shown in Figure 2a‐2c, the corresponding 3D Response surface optimizations of BYWP extraction yield and the independent parameters were plotted, which illustrated the interaction of variables with the uttermost response. The middle values of the three parameters (NaOH addition of 60% (A), ultrasonic power 150 W (B), ultrasonic time 80 min (C)) were used to create the graphs. The BYWP extraction yield first increased then decreased with the increase in NaOH addition, ultrasonic power, and ultrasonic time. Besides, because from the ANOVA results ultrasonic time does not affect significantly the extraction yield by itself, according to the ANOVA results NaOH addition, ultrasonic power, the quadratic factors and the interaction between NaOH concentration and extraction time are affecting significantly extraction yield, with 95% of confidence level. According to the model, the maximum extraction yield of BYWP was 37.54% while the values of corresponding variables were NaOH addition (52.63%), ultrasonic power (143.15 W), and ultrasonic time (86.20 min). Besides, the effects of the other two variables on the BYWP extraction yield were shown in every spot at a time while another variable was maintained as a fixed value.

To improve the operability, the BYWP extraction yield was validated with parameters NaOH addition (53%), ultrasonic power (140 W), and ultrasonic time (86 min). The practical extraction yield was 36.26%, with a relative error of 3.41%. The practical BYWP extraction yield was very close to the predicted yield, which indicated that the model could be used to guide actual practice.

### 3.3 Isolation, purification, and characterization of BYWP

**FIGURE 3:**
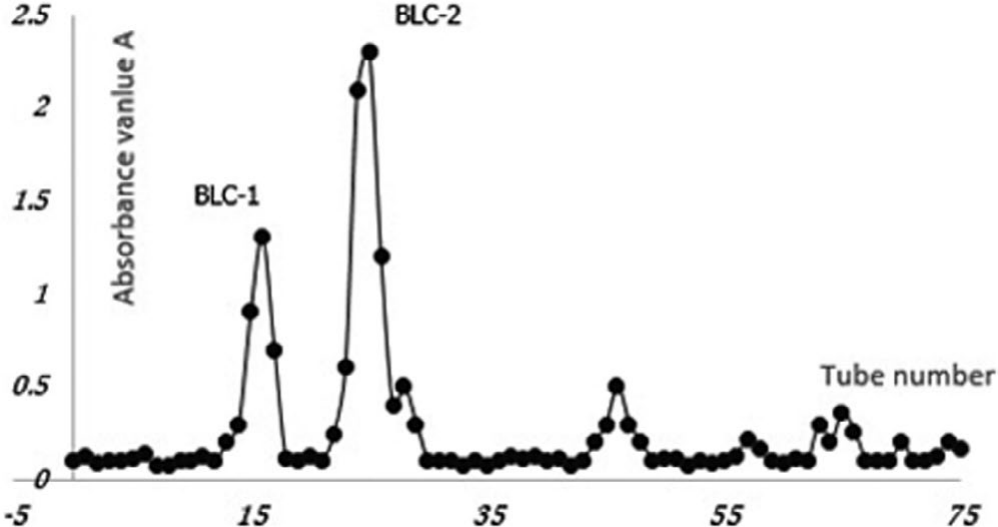
Linear elution figure of pure polysaccharides

After procedures of purification and isolation, the pure BYWP was further isolated and fractionated by DEAE‐52 Sepharose Fast Flow exchange column and Sephadex G‐100 column. The two main fractions were eluted, with a single sharp peak, respectively (Figure 3). They were named BLC‐1 and BLC‐2. The proportions of BLC‐1 and BLC‐2 in pure BYWP were 24.57% and 66.08%, respectively.

**FIGURE 4:**
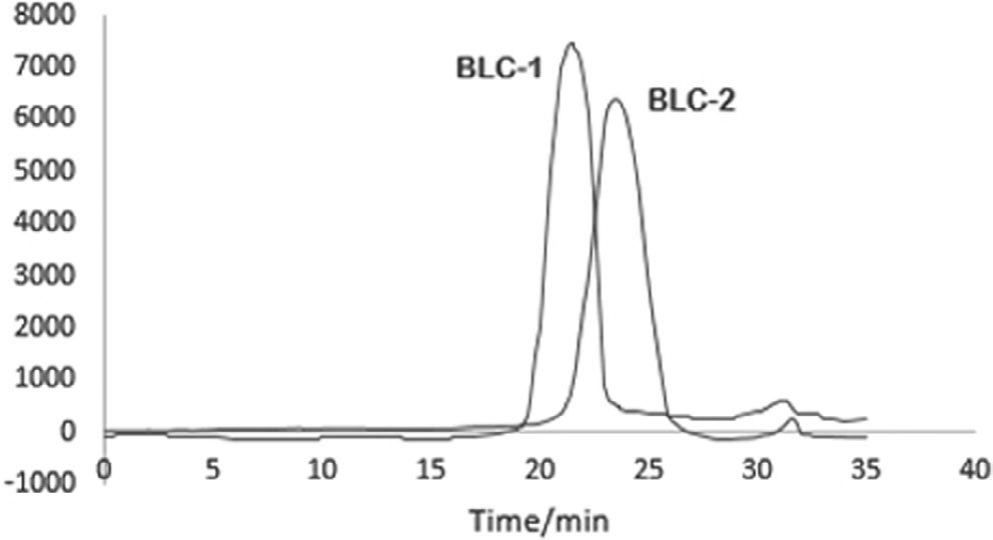
HPGPC chromatograms of BLC‐1 and BLC‐2

The molecular weight of BLC‐1 and BLC‐2 was determined by HP GPC. Two single peaks were observed, which indicated that the components of BLC‐1 and BLC‐2 were pure enough for further analysis (Figure 4). The retention time of BLC‐1 and BLC‐2 was 16.206 min and 20.053 min, respectively. The retention time of BLC‐1 and BLC‐2 were included in the equation, and the molecular weight of BLC‐1 and BLC‐2 were found to be 164.68 kDa and 13.21 kDa.

### 3.4 Analysis of the monosaccharide compositions

**FIGURE 5:**
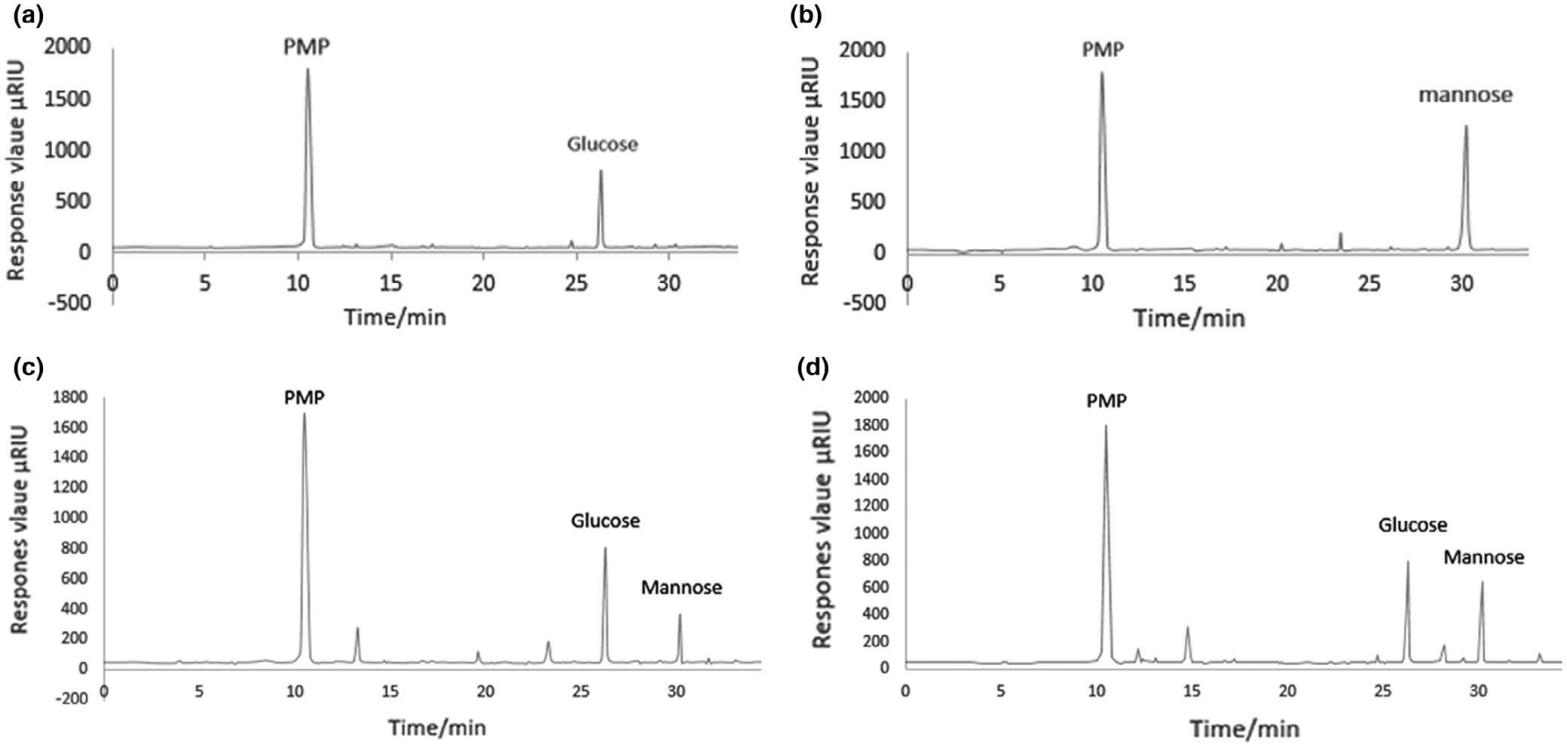
HPLC chromatograms of monosaccharide standards and the BLC‐1, BLC‐2 HPLC chromatogram of glucose (a), HPLC chromatogram of mannose (b), HPLC chromatogram of the BLC‐1 hydrolysate (c), HPLC chromatogram of the BLC‐2 hydrolysate(d)

The prominent peak retention time of glucose and mannose standard samples was 26.289 min and 30.196 min, respectively (Figure 5a and 5b). Figure 5c and 5d showed the chromatographic peak areas of glucose and mannose in BLC‐1 and BLC‐2 components, respectively. The concentrations of glucose and mannose were determined, according to the regression equation. The glucose and mannose contents in BLC‐1 and BLC‐2 compounds were 815.30 μg/ml, 646.07 μg/ml and 1,218.51 μg/mL, 131.11 μg/mL, respectively. The proportions of glucose and mannose in BLC‐1 and BLC‐2 were 47.68%, 39.18%, and 76.59%, 6.86%, respectively.

### 3.5 Effect of BYWP on the feed conversion ratio and diarrhea rate of early‐weaned lambs

No lamb died in the whole experimental period. At the end of the experiment, there were no significant differences in four groups of initial weight and average daily feed intake (*p* >.05), but the Group III and Group IV of final weight, average daily gain, and the feed conversion ratio have been significantly increased, compared with group I (*p* <.05). The addition of 0.3% and 0.5% BYWP significantly increased the final body weight by 0.84% and 0.70% (*p* <.05). The addition of 0.3% and 0.5% BYWP significantly increased the average daily gain by 11.33% and 10.92% (*p* <.05). The addition of 0.3% and 0.5% BYWP significantly decreased the gain feed ratio by 11.97% and 11.11% compared with Group I (*p* <.05).

Besides, the average fecal index and average diarrhea rate were significantly decreased by the addition of 0.3%, 0.5% BYWP compared with the basic milk replacer (*p* <.05), but no statistically significant difference compared with 0.3% BYWP and 0.5% BYWP (*p* >.05) (Table 6). The average fecal score of Group IV and Group V was significantly lower than Group I by 21.74% and 18.32%, respectively (*p* <.05). Compared with Group I, Group III, and Group IV significantly decreased the average diarrhea rate by 31.19% and 25.63%, respectively (Table 7).

**TABLE 7:** Effects of BYWP on feed conversion ratio and diarrhea rate

Items	Group I	Group II	Group III	Group IV
Initial body weight (kg)	7.35 ± 1.13	7.38 ± 1.17	7.36 ± 1.25	7.34 ± 1.69
Final body weight (kg)	12.08 ± 2.01a	12.56 ± 1.52ab	12.96 ± 1.36b	12.92 ± 2.22b
Average daily gain (g/d)	157.19 ± 35.62a	170.00 ± 31.36ab	175.00 ± 11.08b	174.36 ± 13.51b
Average daily feed intake (g/d)	368.23 ± 21.68	365.50 ± 19.85	360.24 ± 26.02	362.93 ± 22.51
Gain feed ratio (F/G)	2.34 ± 0.02b	2.15 ± 0.04ab	2.06 ± 0.02a	2.08 ± 0.03a
Average fecal index	3.22 ± 0.43c	3.27 ± 0.51c	2.52 ± 0.38b	2.63 ± 0.15b
Average diarrhea rate/(%)	36.48 ± 7.90c	33.12 ± 8.33c	25.10 ± 5.20b	27.13 ± 6.29b

### 3.6 Effect of BYWP on intestinal morphology of jejunum and ileum

The changes in intestinal mucosal morphologies for the jejunum and ileum were observed. The atrophy, degeneration, tortuosity, and falling out of the mucosal epithelial cells were observed in the basal milk replacer group. Contrary to the control group, the small intestine epithelium microvilli arranged neatly with regular shape in the addition of 0.5% BYWP group. Besides, we found that the Villus height and Crypt depth of Group IV were distinct longer than those in Group I.

Changes in gastrointestinal Villus height, crypt depth, mucosal thickness, and V/C value in response to the addition of 0.5% BYWP in basic milk replacer were shown in Table 8. The Group III and Group IV of Villus height and Crypt depth in jejunum were significantly higher than Group I by 20.80%, 24.17% and 16.35%, 19.08% (*p* <.05). The Group III and Group IV of Villus height and Crypt depth in ileum were significantly higher than Group I by 14.73%, 18.90%, and 10.74%, 13.80% (*p* <.05). There were no significant differences among four groups of mucosal thickness in the jejunum and ileum (*p* >.05). The Group IV of V/C value in jejunum and ileum were significantly higher than Group I by 4.32% and 4.26% (*p* <.05).

**TABLE 8:** Effects of BYWP on intestinal morphology of jejunum and ileum

Body parts	Items (μm)	Group I	Group II	Group III	Group IV
Jejunum	Villus height	263.82 ± 35.41a	277.18 ± 19.74a	318.69 ± 26.15b	327.58 ± 13.34b
	Crypt depth	189.65 ± 15.93a	198.38 ± 22.29a	220.66 ± 26.10b	225.84 ± 28.82b
	Mucosal thickness	486.58 ± 27.26	495.3 ± 30.52	502.27 ± 23.18	501.18 ± 12.56
	V/C value	1.39 ± 0.22a	1.40 ± 0.21a	1.44 ± 0.07b	1.45 ± 0.10b
Ileum	Villus height	232.98 ± 23.18a	252.25 ± 23.29b	267.29 ± 12.18c	277.01 ± 15.04c
	Crypt depth	165.36 ± 14.11a	174.78 ± 18.64a	183.12 ± 13.15b	188.18 ± 17.52b
	Mucosal thickness	437.64 ± 17.72	436.25 ± 19.02	439.38 ± 27.33	441.76 ± 38.40
	V/C value	1.41 ± 0.31a	1.44 ± 0.18b	1.46 ± 0.08b	1.47 ± 0.05b

### 3.7 Effects of BYWP on antibody and cytokines of the ileum

Table 9 showed the concentrations of antibodies and cytokines in the ileum. The levels of SIgA,IL‐1, and IL‐10 were significantly different in four groups (*p* <.05). Group III and Group IV of SIgA were significantly higher than Group I by 14.53% and 17.78% (*p* <.05). Group III and Group IV of IL‐1 were significantly lower than Group I by 8.05% and 10.40% (*p* <.05). Group III and Group IV of IL‐10 were significantly higher than Group I by 13.03% and 18.31% (*p* <.05).

**TABLE 9:** Effects of BYWP on antibodies and cytokines of the ileum

Items	Group I	Group II	Group III	Group IV
SIgA /( mg/g)	12.66 ± 1.72a	12.99 ± 1.21ab	14.50 ± 2.84b	14.91 ± 1.91b
IgA/( mg/g)	24.90 ± 3.71	26.35 ± 2.69	25.67 ± 5.67	25.18 ± 3.51
IgG/( mg/g)	38.65 ± 3.39	38.84 ± 8.02	39.65 ± 3.21	39.87 ± 8.67
IL−1/( mg/g)	2.98 ± 0.4b	3.00 ± 0.28b	2.74 ± 0.32a	2.67 ± 0.79a
IL−10/( mg/g))	2.84 ± 0.45a	2.49 ± 0.10a	3.21 ± 1.23b	3.36 ± 0.50b
TNF/( mg/g)	3.45 ± 0.28	3.57 ± 0.66	3.38 ± 0.16	3.15 ± 0.78
IFN/( mg/g)	2.69 ± 0.23	2.84 ± 0.17	2.52 ± 0.38	2.46 ± 0.63

### 3.8 Effects of BYWP on the intestinal microflora of the ileum

After the quantitative analysis of the intestinal flora for the ileum, the number of *Lactobacillus, Salmonella,* and *Perfringens* was significantly different in four groups (Table 10). Group III and Group IV of *Lactobacillus* were significantly higher than Group I by 5.80% and 6.55% (*p* <.05). Group III and Group IV of *Salmonella* were significantly lower than Group I by 5.41% and 4.67% (*p* <.05). Group III and Group IV of *Clostridium* *perfringens* were significantly lower than Group I by 8.48% and 11.11% (*p* <.05).

**TABLE 10:** Effects of BYWP on selected intestinal bacteria of the ileum may be more precise

Items	Group I	Group II	Group III	Group IV
*Bifidobacterium*	6.16 ± 0.15	6.02 ± 0.10	6.77 ± 0.13	6.73 ± 0.40
*Lactobacillus*	6.72 ± 0.27a	7.05 ± 0.32ab	7.16 ± 0.28b	7.11 ± 0.24b
*Escherichia coli*	5.44 ± 0.22	5.29 ± 0.14	5.24 ± 0.14	5.21 ± 0.05
*Salmonella*	5.36 ± 0.34b	5.33 ± 0.27b	5.07 ± 0.54a	5.11 ± 0.50a
*Clostridium perfringens*	4.86 ± 0.39b	4.74 ± 0.19ab	4.43 ± 0.11a	4.32 ± 0.23a

## 4 DISCUSSION

Ultrasound‐assisted extraction (UAE) is a green and efficient extraction technology, which has become a very important extraction method for natural polysaccharide extraction. It reported that the water‐soluble polysaccharides from the Acanthus ilicifolius could be extracted by the method of UAE and optimized, in which the optimized conditions were extraction time of 50min, ultrasonic power of 150W, and liquid to solid ratio of 35ml/g (Mtetwa et al., 2020). Besides, the polysaccharides from Rosa roxburghii Tratt fruit have been extracted by UAE technology, and the optimum conditions were ultrasonic power 148W, extraction temperature 78.8°C (Chen and Kan, 2018). In this study, the optimum parameters of ultrasound‐assisted extraction for BYWP were NaOH addition (52.63 g/L), ultrasonic power (143.15 W), and ultrasonic time (86.20 ℃), and we obtained an extraction yield of 37.54%. Invalidating experiments; the practical extraction yield was 36.26%, with a relative error of 3.41% compared with the theoretical maximum extraction yield. This result indicated that the model can be used to guide actual practice and production. In the process of ultrasound‐assisted extraction, the ultrasound power facilitated the disruption of cell walls. A high extraction yield of polysaccharides occurred with the stronger ultrasound power at the early period. However, the acoustic cavitation in a strong ultrasound power yielded a lot of hydroxyl radicals, which could lead to molecular structure decomposition (Koda et al., 2003). Extraction time is another important condition, and the extraction yield of polysaccharides increased with the long extraction time. However, too long extraction time will lead to high temperature, which could increase vapor pressure and the decrease of surface tension within microbubbles, which causes the damping of the ultrasonic wave (Liu et al., 2014). NaOH can destroy the cell wall efficiently, but a high concentration of NaOH could induce the degradation of polysaccharides.

The molecular weight is a fundamental indicator, which could be investigated to imply the polysaccharides bioactivity. It has been reported that most polysaccharides with a molecular weight of 10 to 200 kDa have bioactivity, normally (Cordero et al., 2011). However, Walsh believed that 5 to 10 kDa oligosaccharides possessed antibacterial activity and the 10 to 50 kDa polysaccharide was optimum for increasing intestinal structure (Walsh et al., 2012). Besides, there was evidence suggesting that glucose and mannose are the main biological activity components in polysaccharides (Qiang et al., 2017). In this study, the molecular weights of BLC‐1 and BLC‐2 were 164.68 KDa and 13.21 KDa, respectively, and they accounted for 24.57% and 66.08% of total polysaccharides. Besides, the proportions of glucose and mannose accounted for 47.68%, 39.18%, and 76.59%, 6.86% in BLC‐1 and BLC‐2, respectively. By using these molecular weights and the proportion of glucose and mannose, we can determine that BYWP probably has some biological activities. It has been reported that chestnut kernel polysaccharide was efficiently extracted by using ultrasound‐assisted extraction. The chestnut kernel polysaccharides showed some biological functions, such as antioxidant and bacteriostatic function. UEP1‐1 and UEP2‐1 were the major fractions of chestnut kernel polysaccharide, which the molecular weights were 74.5 KDa and 59.9 KDa, respectively (Tang et al., 2019). In another study, the major polysaccharides were MLP‐1 and MLP‐2 in mulberry leaf polysaccharides, in which the molecular weight of MLP‐1 and MLP‐2 were 222.0 KDa and 93.1 KDa, respectively. The main monosaccharide compositions were mannose, galactose, and mannose (Zhang and Wan et al., 2016).

During the weaning period, the digestive and immune system of early‐weaned lambs are immature, and they have to adapt the drastic change from digestible watery milk to a less digestible solid feed, and the risks of diseases and infection by pathogenic bacteria such as *Salmonella* and *Escherichia coli,* are increased, that makes lambs dead and decreases production performance in sheep industry (Wang et al., 2019). In this study, the addition of 0.3% BYWP in milk replacer achieved the best results in F/G, average fecal index, and average diarrhea rate. Also, there was no significant difference between the addition of 0.3% and 0.5% BYWP in F/G, average fecal index, and average diarrhea rate. This result indicated that the 0.3% and 0.5% BYWP as lamb milk replacer supplement could decrease the feed utilization rate and the rate of diarrhea occurrence in early‐weaned lambs. The more meritorious is that the basal milk replacer supplemented with BYWP did not result in the hidden troubles of antibiotics resistance, drug residues, and environmental pollution. It has been reported that yeast cell wall polysaccharides were a kind of health and safe animal feed additive, and adding 0.2% yeast polysaccharides in the diet could improve the growth performance, reduce the ssdiarrhea rate of weaned pigs, and sucking calves (Dong et al., 2018). Besides, Liu found that 160 mg/kg supplemental chitooligosaccharide reduced the incidence of diarrhea in piglets (Liu et al., 2008). In addition, it has been reported that chitooligosaccharide could promote prevent diarrhea and growth performance in weaned piglets, which was probably mediated by increasing nutrient apparent digestibility, regulating intestinal microbiota, and modulating the production of cytokines and antibodies (Xu et al., 2018).

It has been reported that polysaccharide affects the development of the gastrointestinal tract and impairs the morphology of the small‐intestinal mucosa in humans and several animal models (Yang et al., 2012). The key roles of the villus height, crypt depth, mucosal thickness, and V/C value in processing dietary molecules into available nutrients for the organism make this matter of great importance (Gao et al., 2014). In this study, the tissue slices figures showed that BYWP improved density and the cellularity of the intestinal mucosal cell, and arranged compactly both in jejunum and ileum of early‐weaned lambs. Besides, according to the measurements of tissue slices, the results showed that the villus height, crypt depth, and V/C value were significantly increased by the addition of 0.5% BYWP both in the jejunum and ileum of early‐weaned lambs. It has been reported that 160 mg/kg supplemental chitooligosaccharide increased the villus: crypt ratio in weaned piglets, challenged with *Escherichia* (Liu et al., 2008).

The intestinal mucosal barrier such as tight junction, mucin layer, and the immune system plays an important and essential role in protecting the health of young animals by eliminating antigens, such as pathogenic viruses and bacteria, and toxins (Zhang and Wang et al., 2016; Patra, 2020). The balance among antibody, pro‐inflammatory, and anti‐inflammatory cytokines is of great crucial factors for the health of early‐weaned lambs. Secretory Immunoglobulin A (SIgA) is a major component in intestinal mucosal immune, which could maintain the integrity of the biological barrier, against the kinds of pathogenic bacteria. IL‐1 is an inflammatory cytokine, which could be activated against the invasion of pathogenic bacteria. But the excessive expression of IL‐1 will lead to mucosal cellular damage. IL‐10 is an important anti‐inflammatory cytokine, which could increase the immune tolerance for intestinal protection. In this study, the addition of 0.3% and 0.5% BYWP significantly improved the level of SIgA and IL‐10, but significantly decreased the level of IL‐1, which indicated that the immune response and immune tolerance were enhanced in the intestinal mucosal. It has been reported that supplementation with polysaccharide promoted the secretions of TFF3 and MUC2 in piglets, which could be related to the simultaneous promotion in goblet cell numbers (Hu et al., 2017). Besides, it has been reported that dietary supplementation with 50 mg/kg polysaccharide could significantly reduce the level of cytokines IL‐10 and TGF‐β in jejuna mucosal of weaned piglets (Hu et al., 2017).

At present, the intestinal microbial ecosystem is a virtual endocrine organ, this plays a curial role in body health (Schokker et al., 2018). In this study, the addition of 0.3% and 0.5% BYWP in milk replacer significantly increased the number of *Lactobacillus* but suppressed the growth of *Salmonella* and *Clostridium* *perfringens*, which indicated that BYWP promoted the balance of intestinal flora. It has been reported that Saccharomyces cerevisiae polysaccharides could significantly increase the number of *Lactobacillus*, *Bifidobacterium*, but decrease *Salmonella* and *Escherichia coli* in the cecum of broiler chicken (Jun et al., 2018). Besides, another report showed that the supplementation of yeast mannosan increased the number of *Bifidobacterium*, but decreased the number of *Escherichia coli* in the intestinal of turkey (Karaman et al., 2005).

## 5 CONCLUSION

In this study, the optimal ultrasound‐assisted extraction parameters for BYWP were NaOH addition (52.63%), ultrasonic power (143.15 W), and ultrasonic time (86.20 min). The optimized extraction yield of BYWP was 37.54%. The main components of BYWP were BLC‐1 and BLC‐2. The molecular weight of BLC‐1 and BLC‐2 was 164.68 KDa and 13.21 KDa, respectively, and their proportions in BYWP were 24.57% and 66.08%, respectively. The proportion of glucose and mannose in BLC‐1 and BLC‐2 was 47.68%, 39.18%, and 76.59%, 6.86%, respectively.

During the feeding trial, the results identified that BYWP as a potent natural polysaccharide improved the feed utilization rate and decreased the rate of diarrhea occurrence in early‐weaned lambs. Besides, the supplementation of 0.3% and 0.5% BYWP in basal milk replacer improved the intestinal morphology (villus height, crypt depth, and V/C value) of jejunum and ileum, but there was no significant difference in mucosal thickness. Besides, the addition of 0.3% and 0.5% BYWP significantly improved the level of SIgA and IL‐10, but significantly decreased the level of IL‐1 in the ileum. Finally, the addition of 0.3% and 0.5% BYWP in milk replacer significantly increased the number of *Lactobacillus* but suppressed the growth of *Salmonella* and *Perfringens*.

## CONFLICT OF INTEREST

The authors declare that they do not have any conflict of interest, and the manuscript is approved by all authors for publication.

## ETHICAL APPROVAL

This study's protocols and procedures were ethically reviewed and approved by the Animal Welfare Committee of Shihezi University with the ethical code: A2019‐156–01. Informed Consent: Written informed consent was obtained from all study participants.

## Data Availability

The raw/processed data required to reproduce these findings cannot be shared at this time as the data also forms part of an ongoing study.
